# Cortical substrates and functional correlates of auditory deviance processing deficits in schizophrenia

**DOI:** 10.1016/j.nicl.2014.09.006

**Published:** 2014-10-01

**Authors:** Anthony J. Rissling, Makoto Miyakoshi, Catherine A. Sugar, David L. Braff, Scott Makeig, Gregory A. Light

**Affiliations:** a Department of Psychiatry, University of California San Diego, La Jolla, CA, USA; b Swartz Center for Computational Neuroscience, Institute for Neural Computation, University of California San Diego, La Jolla, CA, USA; c Japan Society for the Promotion of Science, Japan; d VISN-22 Mental Illness, Research, Education and Clinical Center (MIRECC), VA San Diego Healthcare System, Los Angeles, CA, USA; e Department of Psychiatry, University of California Los Angeles, Los Angeles, CA, USA; f Department of Biostatistics, University of California Los Angeles, Los Angeles, CA, USA; g VISN-22 Mental Illness, Research, Education and Clinical Center (MIRECC), Greater Los Angeles VA Healthcare System, Los Angeles, CA, USA

**Keywords:** Schizophrenia, Mismatch negativity, Attention, EEG, Independent component analysis

## Abstract

Although sensory processing abnormalities contribute to widespread cognitive and psychosocial impairments in schizophrenia (SZ) patients, scalp-channel measures of averaged event-related potentials (ERPs) mix contributions from distinct cortical source-area generators, diluting the functional relevance of channel-based ERP measures. SZ patients (*n* = 42) and non-psychiatric comparison subjects (*n* = 47) participated in a passive auditory duration oddball paradigm, eliciting a triphasic (Deviant−Standard) tone ERP difference complex, here termed the auditory deviance response (ADR), comprised of a mid-frontal mismatch negativity (MMN), P3a positivity, and re-orienting negativity (RON) peak sequence. To identify its cortical sources and to assess possible relationships between their response contributions and clinical SZ measures, we applied independent component analysis to the continuous 68-channel EEG data and clustered the resulting independent components (ICs) across subjects on spectral, ERP, and topographic similarities. Six IC clusters centered in right superior temporal, right inferior frontal, ventral mid-cingulate, anterior cingulate, medial orbitofrontal, and dorsal mid-cingulate cortex each made triphasic response contributions. Although correlations between measures of SZ clinical, cognitive, and psychosocial functioning and standard (Fz) scalp-channel ADR peak measures were weak or absent, for at least four IC clusters one or more significant correlations emerged. In particular, differences in MMN peak amplitude in the right superior temporal IC cluster accounted for 48% of the variance in SZ-subject performance on tasks necessary for real-world functioning and medial orbitofrontal cluster P3a amplitude accounted for 40%/54% of SZ-subject variance in positive/negative symptoms. Thus, source-resolved auditory deviance response measures including MMN may be highly sensitive to SZ clinical, cognitive, and functional characteristics.

## Highlights

• Six source clusters contributing to the triphasic auditory deviance response were identified.
• Source resolved responses are sensitive to SZ clinical, cognitive, and function characteristics.
• Source resolved responses accounted for up to half the variance in cognitive and symptom scales.

## 1 Introduction

There is growing evidence that sensory processing impairments contribute to cognitive and psychosocial deficits in schizophrenia (SZ) patients (Braff and Light, 2004; Javitt, 2009). Even when the participant's attention is drawn to another stimulus stream (e.g., here an animated cartoon), average event-related potentials (ERPs) time-locked to presentations of deviant stimuli interspersed in a train of standard tones evoke a response complex dominated by three peaks, labeled mismatch negativity (MMN), P3a, and reorienting negativity (RON), that appears to index preattentive sensory discrimination and attention-related orienting processes (e.g., Näätänen, 1990; Rissling et al., 2012; Rissling et al., 2013). Typically, studies use measures of the difference between responses evoked by infrequent Deviant versus Standard tones in a continuing sequence to avoid contamination by potentials indexing low-level auditory processes common to both responses. Here we refer to this response difference as the ‘auditory deviance response.’

Peak measures of MMN and, to a lesser extent, P3a and RON have emerged as potential biomarkers for improving the understanding and treatment of psychosis (Light and Näätänen, 2013; Perez et al., 2014). Smaller amplitudes of each of these peaks have been consistently identified in chronic (Michie, 2001; Shelley et al., 1991; Umbricht and Krljes, 2005), recent onset (Atkinson et al., 2012; Bodatsch et al., 2011; Brockhaus-Dumke et al., 2005; Hermens et al., 2010; Jahshan et al., 2012; Oades et al., 2006; Oknina et al., 2005; Salisbury et al., 2002; Umbricht et al., 2006) and unmedicated SZ patients (Catts et al., 1995; Kirino and Inoue, 1999; Rissling et al., 2012), with promising utility for preemptive assessment and intervention in at-risk populations (Light and Näätänen, 2013; Nagai et al., 2013; Perez et al., 2014). MMN peak measures, in particular, exhibit high test–retest stability, allowing their use in repeated measure designs. Further, MMN measures have often-reported relationships to cognition and psychosocial function (Light and Braff, 2005a; Light et al., 2012; Nagai et al., 2013), and EEG data collection during passive oddball paradigms is comfortably tolerated even by highly impaired or symptomatic individuals. Although the cortical sources of MMN are not well defined, pharmacologic and animal model studies show MMN peak amplitude as measured on the frontocentral scalp is a sensitive index of NMDA (Ehrlichman et al., 2008; Gil-da-Costa et al., 2013; Javitt et al., 1996; Lavoie et al., 2008; Nagai et al., 2013; Nakamura et al., 2011) and nicotinic receptor functioning (Preskorn et al., 2014).

As part of an effort to develop a stronger neuroscientific basis for psychiatric assessment and care, separate expert consensus panels convened by the Institute of Medicine (Pankevich et al., 2011) and by Cognitive Neurosciences Treatment Research to Improve Cognition in Schizophrenia (CNTRICS) have supported the use of MMN measures in next-generation approaches to understanding and treating psychotic illnesses. CNTRICS highlighted MMN as a “mature” biomarker ready for immediate incorporation into multi-site trials (Butler et al., 2012), contributing to a view of MMN peak amplitude as a “breakthrough biomarker” (Belger et al., 2012; Light and Näätänen, 2013).

Despite enthusiasm for MMN as a candidate biomarker that can inform future therapeutic studies of schizophrenia, the majority of clinical studies typically focus on a single frontocentral electrode (Fz) at which both peak amplitudes and patient deficits tend to be the largest (e.g., Light et al., 2012). Many investigators have productively applied multi-sensor EEG recording and event-related trial averaging to investigate the neural architecture underlying normal and impaired sensory processing in SZ, demonstrating the existence of at least two cortical generator areas in or near supratemporal and frontal cortex (Rinne et al., 2000; Takahashi et al., 2012).

However, conventional approaches to EEG analysis do not access the full wealth of information about brain dynamic processes contained in scalp EEG signals. As is well known, raw EEG data includes (and can often be dominated by) non-physiological noise (e.g., 60-Hz line and electrode movement artifacts) and by potentials contributed by non-brain physiological processes (e.g., by scalp and neck muscle tension, eye blinks and saccades), particularly in clinical samples. Brain-generated contributions to EEG signals are predominantly the sum of far-field potentials arising from areas of emergent, locally coherent cortical field activity. The well-established biophysics of brain volume conduction confirm that nearly every scalp electrode sums potentials from nearly every active cortical source (Acar and Makeig, 2013). Thus, currents recorded at scalp channels do not flow directly upwards from the underlying cortex, a common misperception dubbed the topographic fallacy (Coles, 1989). The difficulty in deriving accurate estimates of the brain sources of the recorded scalp potentials is the primary reason that in recent decades EEG has been denigrated as being at best a low-resolution brain imaging modality despite its superior time resolution and other desirable qualities (Onton and Makeig, 2006).

In contrast, application of independent component analysis (ICA) to *unaveraged* EEG data allows spatiotemporal separation of brain and non-brain (artifact) sources (Delorme et al., 2012; Makeig et al., 2004; Makeig et al., 1997; Makeig et al., 2002), capitalizing on information contained within the *whole* EEG data collected during the task session for more precise identification and quantification of cortical areas contributing to the data and to measures derived from it including the auditory deviance response. While fMRI research has widely benefited from application of ICA decomposition, until recently its computational demands and novelty relative to long-standard ERP analysis methods may have limited their natural extension to source-resolved EEG investigations in clinical populations (Calhoun et al., 2010; Demirci et al., 2009; McLoughlin et al., 2014a).

Theoretically, more direct measures of the distinct contributions of cortical areas producing the auditory deviance response should exhibit more robust relationships to group and individual subject illness-related symptom and function differences than measures of scalp-channel ERPs that sum all the source contributions. This study aimed to identify the primary sources of the auditory deviance response complex in SZ and non-psychiatric comparison subjects (NCS), and to explore whether source-level ERP measures are more sensitive than standard scalp-channel measures to clinical, cognitive, and functional SZ characteristics.

## 2 Materials and methods

### 2.1 Participants

Participants included 47 NCS and 42 SZ patients (Table 1, Table 2). There were additional 20 datasets recorded from SZ patient family members; these datasets were not entered into the statistical comparisons reported here. SZ patients were recruited from community residential facilities and via clinician referral. All patients were clinically stable. Clinical symptoms were assessed with the Scale for the Assessment of Negative Symptoms (SANS; Andreasen, 1984) and the Scale for the Assessment of Positive Symptoms (SAPS; Andreasen, 1984). Most were prescribed combinations of psychotropic and non-psychotropic medications with a single second-generation antipsychotic medication (*n* = 29) being the most common, followed by first-generation antipsychotic medications (*n* = 3), a combination of the first and second generation medications (*n* = 8) or no medication for at least 1 month prior to testing (*n* = 2). Audiometric testing (Saico, Assens, Denmark; Model SCR2) was used to ensure that participants had normal hearing in both ears and could detect 45-dB sound pressure level tones at 500, 1000, and 6000 Hz.

NCS were recruited through Internet advertisements. Exclusionary factors included evidence of Axis I psychiatric and neurological disorders other than schizophrenia, Cluster A personality disorders (SCID for Axis II disorders), head injury, stroke, substance abuse (except tobacco) or a history of Axis I disorders in first degree relatives of NCS as determined by the Family Interview for Genetic Studies (Maxwell, 1992).

All participants were assessed on their capacity to provide informed consent. After subjects were given a detailed description of their participation in the study, written consent was obtained via methods approved by the University of California San Diego (UCSD) institutional review board (No. 071831). Urine toxicology screens were used to rule out recent drug use. All participants were evaluated via the Structured Clinical Interview for DSM-IV (First et al., 1995, 1996).

### 2.2 Stimuli and procedures

A duration-deviant auditory oddball paradigm was employed following our established procedures (Kiang et al., 2009; Light and Braff, 2005a; Light et al., 2007; Light et al., 2012; Rissling et al., 2012; Rissling et al., 2010; Rissling et al., 2013). Subjects were presented with binaural tones (1-kHz, 85-dB, with 1-ms rise/fall, stimulus onset-to-onset asynchrony 500 ms) via insert earphones (Aearo Company Auditory Systems, Indianapolis, IN; Model 3A). Standard (*p* = 0.90, 50-ms duration) and Deviant (*p* = 0.10, 100-ms duration) tones were presented in pseudorandom order with a minimum of 6 Standard stimuli presented between each Deviant stimulus. During the approximately 20-min session, participants watched a silent cartoon video. Participants were instructed to attend to the video as they might be asked to answer questions about it at the end of the session.

### 2.3 Electroencephalographic (EEG) recording, processing, and analysis

**Fig. 1:**
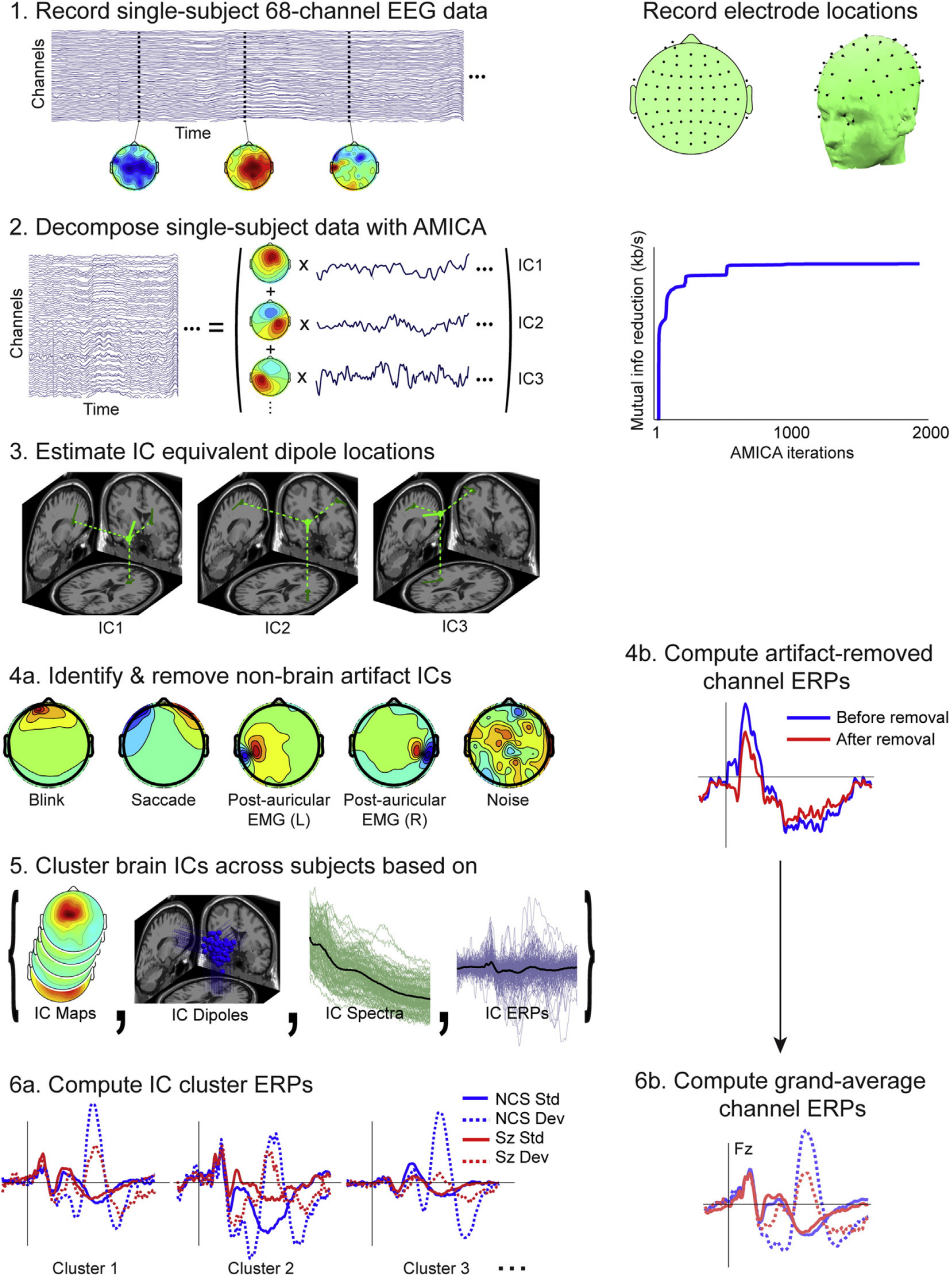
Schematic of the EEG data-processing pipeline, with sample results of the steps in the data analysis: (1) recorded single-subject, 68-channel, raw EEG data plus typically complex EEG scalp maps at three sample time points; (right) the standard locations of the 68 scalp channels in 2-D and 3-D views. (2) Decomposition of single-subject data by adaptive mixture ICA into spatially-fixed projections (scalp maps) of source processes with maximally independent component (IC) time courses (traces); (right) increasing mutual information reduction achieved by the iterative decomposition process. (3) Estimates of single equivalent dipole model locations and orientations for 3 independent component (IC) processes (4a). Identification and removal from further processing of characteristic non-brain artifact ICs (4b). Computed artifact-removed channel ERPs. (5) Brain source ICs clustered across subjects based on their scalp maps, dipole locations, mean power spectra, and auditory ERPs. (6) Source-resolved ERPs for three IC clusters most strongly contributing to the channel ERPs.

Fig. 1 gives a schematic overview of the analysis process. In brief, we ran independent component analysis over each subject dataset and found the best-fitting single equivalent dipole model for each independent component (IC). To enable group-level analysis, we used k-means to find clusters of equivalent ICs across subjects based on IC equivalent dipole locations, ERP time courses, mean log power spectra, and scalp maps, obtaining 20 IC clusters allowing identification of IC source-resolved EEG processes occurring in response to processing of auditory deviance.

### 2.4 EEG data collection

EEG data were continuously digitized at a rate of 500 Hz (nose reference, forehead ground) using an 80-channel Neuroscan system (Neuroscan Laboratories, El Paso, Tex). All scalp channel impedances were brought below 4 kΩ. The system acquisition band pass was 0.5–100 Hz. To prepare data for ICA decomposition and subsequent IC measure computation, data were preprocessed using EEGLAB v11.0.1.0b running under Matlab R2012a (The MathWorks, Natick, MA, USA).

### 2.5 EEG data preprocessing

A 1–100 Hz band pass filter was applied to the continuous EEG data and occasional periods of non-stereotyped artifact were removed to reduce non-stationarity of the data and to improve performance of the subsequent ICA decomposition. The channel montage was based on standard positions in the International 10–5 electrode position system fit to the MNI template head used in EEGLAB (Fig. 1, panel 1).

To improve subsequent ICA decomposition, rejection of abnormal data periods was performed on 500-ms time windows beginning at stimulus onsets. Rejection thresholds for abnormal amplitudes were ±150 µV, and for the subsequent data improbability test (Delorme et al., 2007) >5 SD for each channel and >2 SD for all channels. Time windows containing data points that exceeded more than one of these criteria were discarded. As a result, a mean of 1997 standard trials (SD = 239, range 1425–2552) and a mean of 215 target trials (SD = 24, range 167–278) remained for the NCS group, and a mean of 1999 standard trials (SD = 220, range 1335–2537) and a mean of 218 target trials (SD = 24, range 163-276) remained for the SZ group.

### 2.6 Independent component analysis

The continuous raw EEG data were decomposed using Adaptive Mixture Independent Component Analysis (AMICA) (Palmer et al., 2006; Palmer et al., 2008). The AMICA algorithm was chosen based on its superior performance relative to many other blind source separation approaches both in minimizing remaining mutual information between the maximally independent source processes and in maximizing the number of such processes compatible with a single cortical source area (Delorme et al., 2012). This produced 68 independent components (ICs) per dataset, giving 3196 ICs for the 47 NCS and 2856 ICs for the 42 SZ subjects. In the early iterations of AMICA decomposition, data points that did not fit the model (threshold SD = 5) were excluded from AMICA computation using AMICA *do_reject* option, which was repeated five times after iterations 4, 7, 10, 13, and 16. AMICA convergence was assured by performing 2000 iterations, during which mutual information reduction achieved by the channels-to-ICs linear transformation reached its asymptote (Fig. 1, panel 2).

### 2.7 Independent component localization

For each IC, the 3-D location of the best-fitting equivalent current dipole was estimated using DIPFIT 2.2 (EEGLAB plug-in using Fieldtrip toolbox functions, developed by Robert Oostenveld) using a Montreal Neurological Institute (MNI) template head model. The close resemblance of the projection patterns of many EEG independent component (IC) processes to the projection of a single equivalent current dipole is compatible with an origin in (partially) coherent local field activity across a single cortical area or patch (Delorme et al., 2012). Since the ‘dipolarity’ of the IC scalp maps has been shown to reflect quality of decomposition (Delorme et al., 2012), ICs whose equivalent dipole model when projected to the scalp accounted for less than 85% of the IC scalp map were excluded from further analyses. Similarly, ICs whose equivalent dipoles that were located outside the brain were also excluded, these restrictions retaining 1009 ICs in NCS (31%, 21.5 per subject) and 809 ICs (29%, 19.3 per subject) in SZ (Fig. 1, panel 3). Example scalp maps of ICs rejected for lack of ‘dipolarity’ or equivalent dipole location outside the brain are shown in Fig. 1, panel 4a with labels indicating their eye movement, electromyographic (EMG), or (not further assignable) noise origins.

### 2.8 Scalp-channel ERPs

To compare the sensitivity, selectivity and associations of the source resolved ERPs to clinical, cognitive, and functional measures against measures from traditional scalp-channel ERPs, the scalp-channel data (following removal of the scalp projections of identified non-brain IC processes) were computed using conventional trial averaging procedures. After removal from the channel data of the scalp projections of ICs accounting for non-brain artifacts, standard stimulus-locked ERPs were computed for each subject and channel (see example in Fig. 1, panel 4b). Grand-average channel ERPs were then computed for each subject group and stimulus category (Fig. 1, panel 6b).

### 2.9 Independent component clustering

IC activity and brain location measures used for IC clustering were as follows: equivalent dipole location (dimensions: 3, relative weighting: 10), scalp map (dimensions: 7, weighting: 3), mean log power spectrum (3–50 Hz range, dimensions: 5, weighting: 2), and the Standard and Deviant tone ERPs (0–500 ms range relative to stimulus onset, dimensions: 5, weighting: 1) (Fig. 1, panel 5). To emphasize spatial compactness of IC source clusters we gave the highest weight to IC equivalent dipole locations (10) and scalp maps (3). In STUDY clustering equivalent dipole locations do not retain dipole orientation whose variations across individuals, produced by individual differences in gyrification patterns, can cause considerable variations in scalp topographies of IC projections, even those with completely equivalent source locations, which may occur. We gave larger weight to dipole location, because it can therefore be more robust than the scalp map (Also, its dimension is limited to 3, whereas scalp maps are reduced by principal component analysis to their principal subspace, here with dimension 7). We gave a higher weight to power spectra (2) than to ERPs (1) because power spectra are more sensitive to non-EEG artifacts.

Our experience has suggested that (unless the number of subjects and channels is quite large) it may be better to limit the number of IC clustering dimensions to 20 or less^1^. Therefore, in the current analyses we chose 20 clusters to give a sufficient margin and also to obtain intuitively comprehensive results, in particular, allowing the maximum chance for each cluster to include one IC from each subject, since on average 20.4 ICs per subject were retained for clustering.

Based on metric distance between IC locations in the above location and activity-measure vector space, IC clustering was performed using the k-means method in EEGLAB applied to the IC from SZ, NCS, and SZ family members, generating clusters accounting for distinct brain EEG source areas as well as non-brain EMG, electrooculographic (EOG), and electrocardiographic (ECG) source signals that were also separated by ICA decomposition of the recorded data. These clusters were further inspected manually to check consistency, and some manual adjustments were performed without regard to subject group to ensure cluster homogeneity. This included rejecting outlying ICs in some clusters by visual inspection of their scalp maps, etc., and splitting a large frontal medial cluster into superior and inferior frontal sub-clusters, giving 21 clusters in all. On average, NCS contributed 15.5 ICs to these clusters (±3.7, standard deviation) and SZ subjects (excluding one outlier subject who made no contribution) contributed 14.2 ICs (±3.9, SD). The median number of clusters in which NCSs were represented was 12 (±2.3, SD), whereas SZ subjects contributed to 11 clusters (median; ±2.4, SD). Next, clusters identified by their scalp maps, dipole locations, and mean power spectra as comprised of non-brain artifact component processes (eye movements, line noise, muscle activity, ECG, etc.) were excluded from further analysis.

### 2.10 Constructing an EEGLAB STUDY structure

To perform measure-based IC clustering to identify similar contributing ICs across participants and groups, a three-group (NCS, SZ, Family) × two-stimulus type (Standard, Deviant) EEGLAB STUDY data structure was created. For the present analysis, SZ family group data were excluded from the statistical STUDY design, giving a 2 × 2 STUDY design (2 groups by 2 stimulus types). The EEGLAB study structure allowed use of EEGLAB graphics to visualize grand mean IC cluster measures and their significant group differences.

### 2.11 Cortical source ERP contributions

**Fig. 2:**
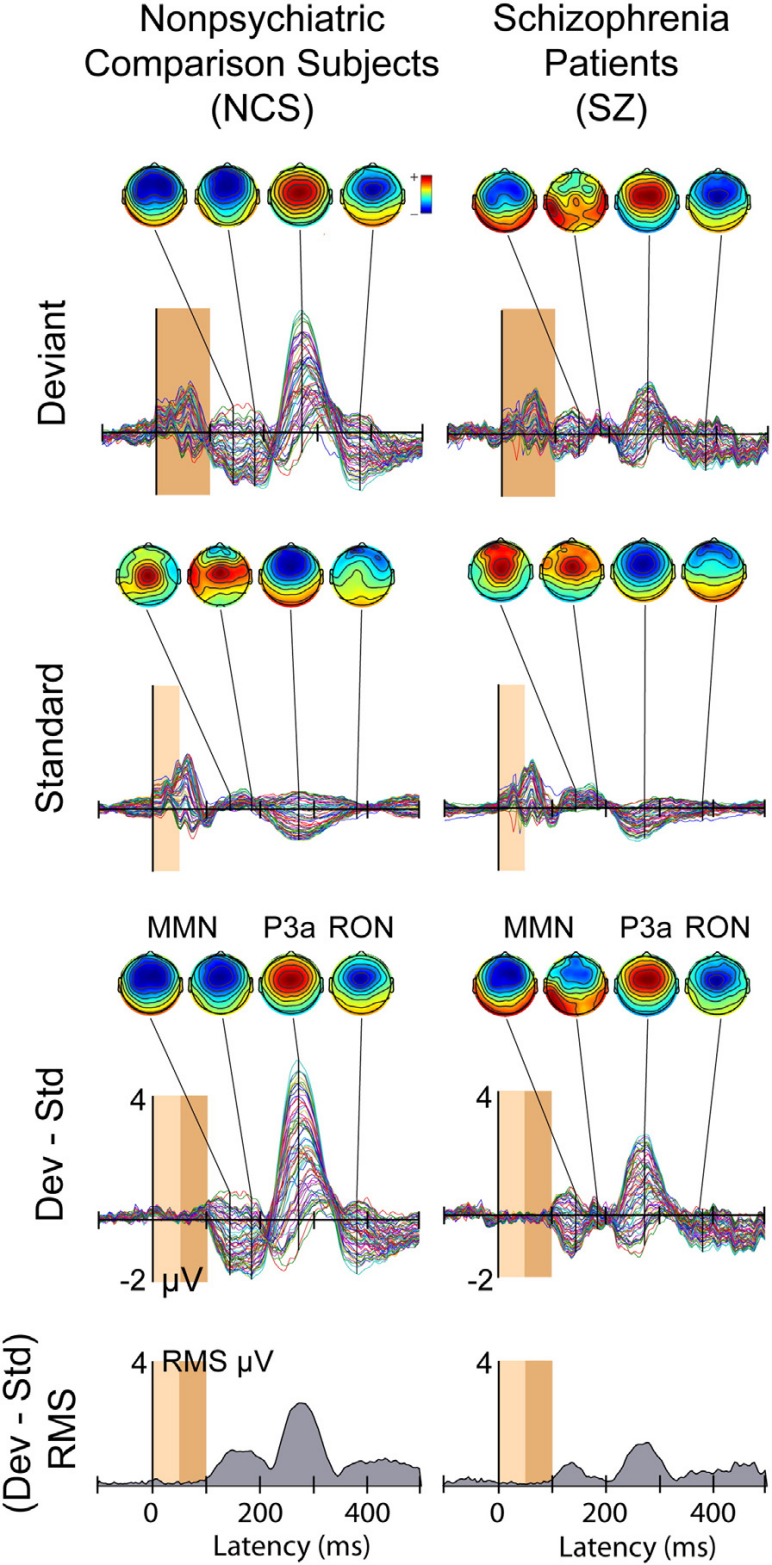
(1st and 2nd rows) group mean Standard and Deviant stimulus ERP waveforms for 68 all scalp channels, after removal of non-brain artifact ICs (3rd row). The (Deviant–Standard) response difference or *auditory deviance response* for the (left) NCS and (right) SZ groups. The deviance response waveforms are dominated by the triphasic MMN–P3a–RON peak sequence, smaller in SZ participants (right column). Scalp maps show the scalp topographies at the group-mean (early and late) MMN, P3a, and RON peak latencies. The bottom row plots the time course of RMS amplitude in the deviance responses (3rd row). The dark brown window shows the duration of the deviant stimuli (100 ms, *p* = 0.10) and the light brown window the duration of the standard stimuli (50 ms, *p* = 0.90).

The contribution of each IC in each source cluster to the subject auditory deviance response was computed by subtracting the source-resolved ERP time locked to Standard tones from the ERP time-locked to Deviant tones. To compute trial-averaged ERPs for each IC, the IC activity data were segmented into epochs from −100 to 500 ms relative to stimulus onsets. After averaging epochs time-locked to Standard and Deviant stimuli, respectively, mean activities in the ERP baseline periods (defined as from −100 to 0 ms relative to stimulus onset) were subtracted from the mean ERPs. Note the importance of subtracting ERP epoch baselines after performing ICA decomposition (Groppe et al., 2009). Grand-average IC cluster ERPs for each group and stimulus category were then computed (Fig. 1, panel 6a). Here we focused on the IC clusters contributing most strongly to the scalp auditory deviance response (across all scalp channels), based on the percent variance accounted for (*pvaf*) by each source cluster across the 500 ms window following stimulus onset in the all-subjects grand average auditory deviance response. Talairach coordinates of cluster equivalent dipole centroids were computed and used to locate and visualize the most strongly contributing clusters (Lancaster et al., 2000; Fig. 2).

### 2.12 Source-resolved ERPs

MMN, P3a and RON ERP peak amplitude and latency measures were computed for IC component processes in the contributing cortical source clusters. Peak amplitude and latencies were inspected following automated peak scanning procedures in the (MMN; 140–240), (P3a; 220–340) and (RON; 310–460) temporal windows. Once the ERP peak latencies were established, their amplitudes were measured using EEGLAB extension *std_ErpCalc* as the mean voltage in the 20 ms surrounding this fixed latency.

### 2.13 Cognitive assessments

The WRAT3 reading subtest was used to assess single word reading ability. Verbal memory was assessed via the California Verbal Learning Test II (CVLT-II) using the List A 1–5 total score to assess immediate verbal memory and long-delay free recall to measure the verbal recall of words during a 20-min interval (delayed verbal memory). Perseverative responses on the Wisconsin Card Sorting Test-64 (WCST-64) were used to assess executive functioning (Heaton, 1993). Performance on the Letter–Number Sequencing (LNS) test was used to assess auditory attention via the immediate on-line storage and repetition of auditory information (forward condition) as well as working memory via manipulation and retrieval of stored information (reordering condition) (Gold et al., 1997; Perry et al., 2001; Wechsler, 1997).

### 2.14 Assessment of functional capacity

Patients' functional capacity was assessed with the UCSD Performance Based Skills Assessment (UPSA; Patterson et al., 2001). The UPSA directly measures functional skills, using standardized tasks that are commonly encountered in everyday situations and considered necessary for independent community living including: general organization and planning, finance, communication, transportation, and household chores.

### 2.15 Assessment of psychosocial functional status

A modified Global Assessment of Functioning (GAF) Scale (Hall, 1995) was used for assessing participants' overall level of functional status across psychological, social, and occupational domains via an anchored measure in accordance with previously published methods (Hall, 1995; McGlashan et al., 2003; McGlashan et al., 2006). In addition to the GAF, the Scale of Functioning was used to assess psychosocial functional status in domains of independent living, social, and instrumental functioning (Rapaport et al., 1996).

### 2.16 Statistical analyses

To determine how scalp averaged ERP latencies and amplitudes differed as a function of Group (NCS, SZ), one-way ANOVAs were applied to MMN, P3a, and RON peak measures. To determine how the key dependent variables differed as a function of Group (NCS, SZ) and cortical source cluster, general linear mixed models (GLMMs) were used. GLMMs allow for flexible covariance structures to account for within-subject correlations, easily accommodate covariates of all types and automatically handle missing data, producing unbiased estimates as long as the observations are missing at random. Models were fit using source-resolved MMN, P3a, and RON ERP peak amplitudes and latencies as the outcomes. Group (NCS, SZ) was included as a between-subject factor, Cluster as the within-subject factor. A Group-by-Cluster interaction term was included to obtain a fully parameterized primary model. Age was included as a covariate. All models were fit using SAS routine PROC Mixed (SAS Institute, Cary, NC) using an unstructured covariance matrix to provide maximum flexibility.

Significant interactions were followed with appropriate pair-wise contrasts within the primary model framework to further characterize the patterns of results. All post hoc comparisons were two-tailed with α-level = 0.05. Spearman's non-parametric correlation coefficients were used to examine the relationships between the ERP peaks with clinical, neurocognitive, and functional measures (shown in Table 3, Table 4 and Inline Supplemental Table 1, Inline Supplemental Table 2).

Inline Supplemental Tables 1 and 2

**Table 1:** Latency correlations. Summary of associations among scalp electrode Fz and source-resolved ERP latencies with neurocognitive variables in non-psychiatric comparison subjects. Correlations shown in bold exceed two-tailed Bonferroni significance level adjustments (Fz; α = 0.05/36 = 0.002; *r*^2^ values > 0.26), (source-resolved ERPs; α = 0.05/216 = 0.0002; *r*^2^ values > 0.32). Number of significant correlations: (Fz) uncorrected = 2, Bonferroni = 2, source resolved ERPs uncorrected = 6, Bonferroni = 1.

	ERP	*r*^2^
Scalp electrode (Fz)		
Working memory (LNS reorder)	**MMN**	**0.24**
Working memory (LNS reorder)	**P3a**	**0.27**
R superior temporal		
Working memory (LNS reorder)	MMN	0.27
Executive functioning (WCST)	RON	0.29
R inferior frontal		
**Auditory attention (LNS forward)**	**RON**	**0.52**
Ventral mid-cingulate		
---n/a---		
Anterior cingulate		
Immediate verbal memory (CVLT)	P3a	0.15
Medial oribitofrontal		
---n/a---		
Dorsal mid-cingulate		
Executive functioning (WCST)	P3a	0.24
Executive functioning (WCST)	RON	0.24

Inline Supplemental Table 1

Inline Supplemental Tables 1 and 2

**Table 2:** Amplitude correlations. Summary of associations among scalp electrode Fz and source-resolved ERP amplitudes with neurocognitive variables in non-psychiatric comparison subjects. Correlations shown in bold exceed two-tailed Bonferroni significance level adjustments (Fz; α = 0.05/36 = 0.002; *r*^2^ values > 0.26), (source-resolved ERPs; α = 0.05/216 = 0.0002; *r*^2^ values > 0.32). Number of significant correlations: (Fz) uncorrected = 2, Bonferroni = 2, source resolved ERPs uncorrected = 6, Bonferroni = 2.

	ERP	*r*^2^
Scalp electrode (Fz)		
Working memory (LNS reorder)	**MMN**	**0.24**
Working memory (LNS reorder)	**P3a**	**0.27**
R Superior temporal		
**Immediate verbal memory (CVLT)**	**P3a**	**0.35**
R inferior frontal		
---n/a---		
Ventral mid-cingulate		
---n/a---		
Anterior cingulate		
Immediate verbal memory (CVLT)	RON	0.17
Delayed verbal memory (CVLT)	RON	0.15
Dorsal mid-cingulate		
Verbal IQ (WRAT Reading)	MMN	0.19
**Verbal IQ (WRAT Reading)**	**RON**	**0.32**
Working memory (LNS reorder)	P3a	0.25

Inline Supplemental Table 2

To minimize the likelihood of Type I errors that could occur from performing multiple statistical tests, correlations were deemed significant only if observed associations accounted for more than 10% of the variance. We also tested whether the number of significant correlations observed exceeded what would be expected by chance alone, stratified by magnitude of association. Counts of number of observed correlations as well as those that would be expected by chance alone are shown in Table 5 and Supplemental Table 3. Bonferroni adjustments (2-tailed) were performed to correct for multiple comparisons. For corrections involving traditional Fz scalp ERP difference wave measures, the adjusted significance threshold was α = 0.05/30 (3 peaks × 10 clinical variables) = 0.0016; with a sample of size *n* = 42, *r*^2^ values larger than 0.22 were considered significant. For correlations with source-resolved difference-ERP measures, the adjusted significance threshold was α = 0.05/180 (6 sources × 3 peaks × 10 clinical variables) = 0.00027; *r*^2^ > 0.28.

Inline Supplemental Table 3

**Table 3:** Summary of expected (based on chance alone) and observed correlations stratified by magnitude of *r*^2^effect sizes in non-psychiatric comparison subjects. Bonferroni adjusted critical *p*-values (2-sided) for traditional ERP averaging at electrode Fz and the combined 6 sources pooled across MMN, P3a and RON ERPs are shown.

*r*^2^	Adjusted critical *p*-value	Expected # significant correlations		Observed # significant correlations (amplitude)		Observed # significant correlations (latency)	
Traditional ERP	Source resolved ERP	Traditional ERP	Source resolved ERP	Traditional ERP	Source resolved ERP
>10%	.037	0.89	5.34	2.0	6.0	2.0	6.0
≥20%	.0023	0.06	0.33	2.0	3.0	2.0	5.0
≥30%	.00012	0.003	0.02	0.0	1.0	0.0	1.0
≥40%	.000004	0.0001	0.0006	0.0	0.0	0.0	1.0
≥50%	.00000008	0.000002	0.00001	0.0	0.0	0.0	1.0

Inline Supplemental Table 3

## 3 Results

### 3.1 Scalp-channel response peak amplitudes

Fig. 2 shows all-channels plots of the grand average Standard, Deviant, and response difference ERPs for the NCS and SZ groups, the difference responses exhibiting the expected MMN, P3a and RON ERP peak features. For both groups, maximal peak amplitudes occur at scalp channel Fz (heavy line). For later comparison with source projections, the time courses of root mean-square (RMS) ERP amplitude (across all channels) are shown below the ERP waveforms.

### 3.2 Primary contributing IC clusters

The six primary cortical source clusters contributing to the (Deviant−Standard) auditory deviance response were the same in both groups, and neither group dominated any of the clusters beyond what would be expected by chance alone (see Table 6). On average, just over 55% of each subject group contributed to each cluster, and for both groups each contributing subject contributed on average just over 1.3 ICs to each cluster. On average, SZ subjects contributed 4.1 (±1.9 SD) ICs to 3.1 (±1.3 SD) of the 6 contributing clusters, while NCS subjects contributed 3.7 (±1.8 SD) ICs to 2.9 (±1.3 SD) of these clusters.

**Fig. 3:**
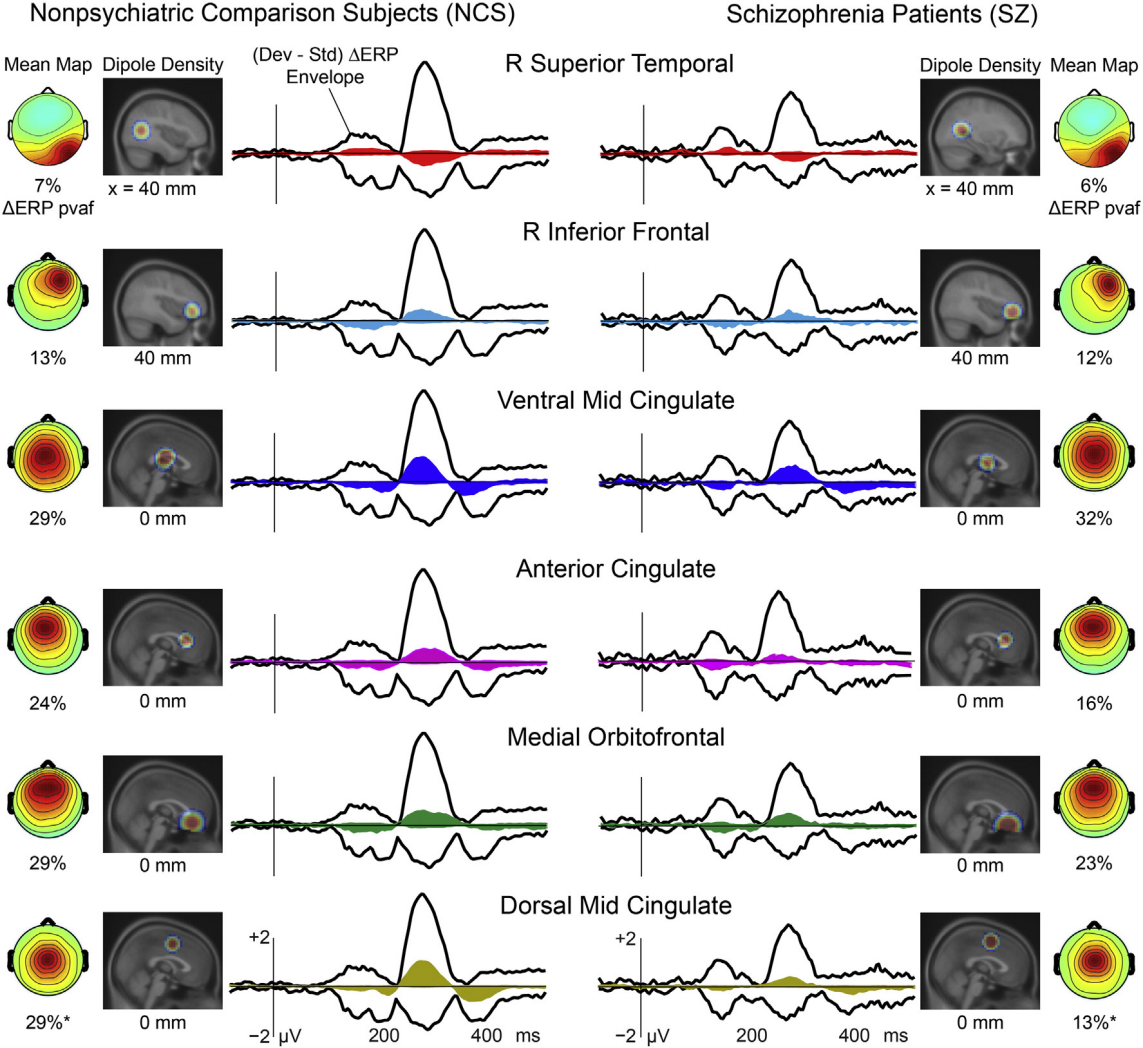
The six IC source clusters contributing most strongly to the auditory deviance response by percent variance accounted for (pvaf). Vertical black lines indicate tone onset. The black outer traces show the envelope (most positive and negative channel values) of the group mean scalp-channel deviance response (after artifact rejection); colored envelopes indicate the (min, max) envelopes of the summed scalp-channel contributions of the independent components in each IC source cluster. The (left and right) dipole density maps indicate, on a relevant sagittal MNI (Montreal Neurological Institute) template brain slice, the locations of each source cluster for the two groups. Scalp maps (far left and right) show the peak scalp topography of the summed source cluster ERP projection, and (below this) the percent variance accounted for (pvaf) in the scalp-channel deviance response by the IC cluster contribution. Note the near-equal pvaf in NCS and SZ for three source clusters (Clusters 1–3), and the smaller pvaf contribution in SZ participants for three other frontal midline source clusters (Clusters 4–6).

Equivalent model dipoles for the six clusters were centered in or near R Superior Temporal, R Inferior Frontal, Ventral Mid-Cingulate, Anterior Cingulate, Medial Orbitofrontal, and Dorsal Mid-Cingulate cortex. Fig. 3 shows their scalp topographies, current dipole densities and percent variance (of the response difference) accounted for, here separated into IC cluster subsets for the SZ and NCS subject groups, respectively. *T*-tests showed that the numbers of ICs from each group did not differ significantly for any of the clusters (*p* > 0.05). Asterisks (or NS = not significant) near the ‘pvaf’ percentages indicate the significance of the group difference. SZ patients produced visibly smaller auditory deviance response contributions from 5 of the 6 IC clusters, and proportionally smaller (pvaf) contributions from several of these clusters, most strongly (and significantly, at α = 0.05) from the dorsal mid-cingulate cluster.

### 3.3 Auditory deviance response group differences

**Fig. 4:**
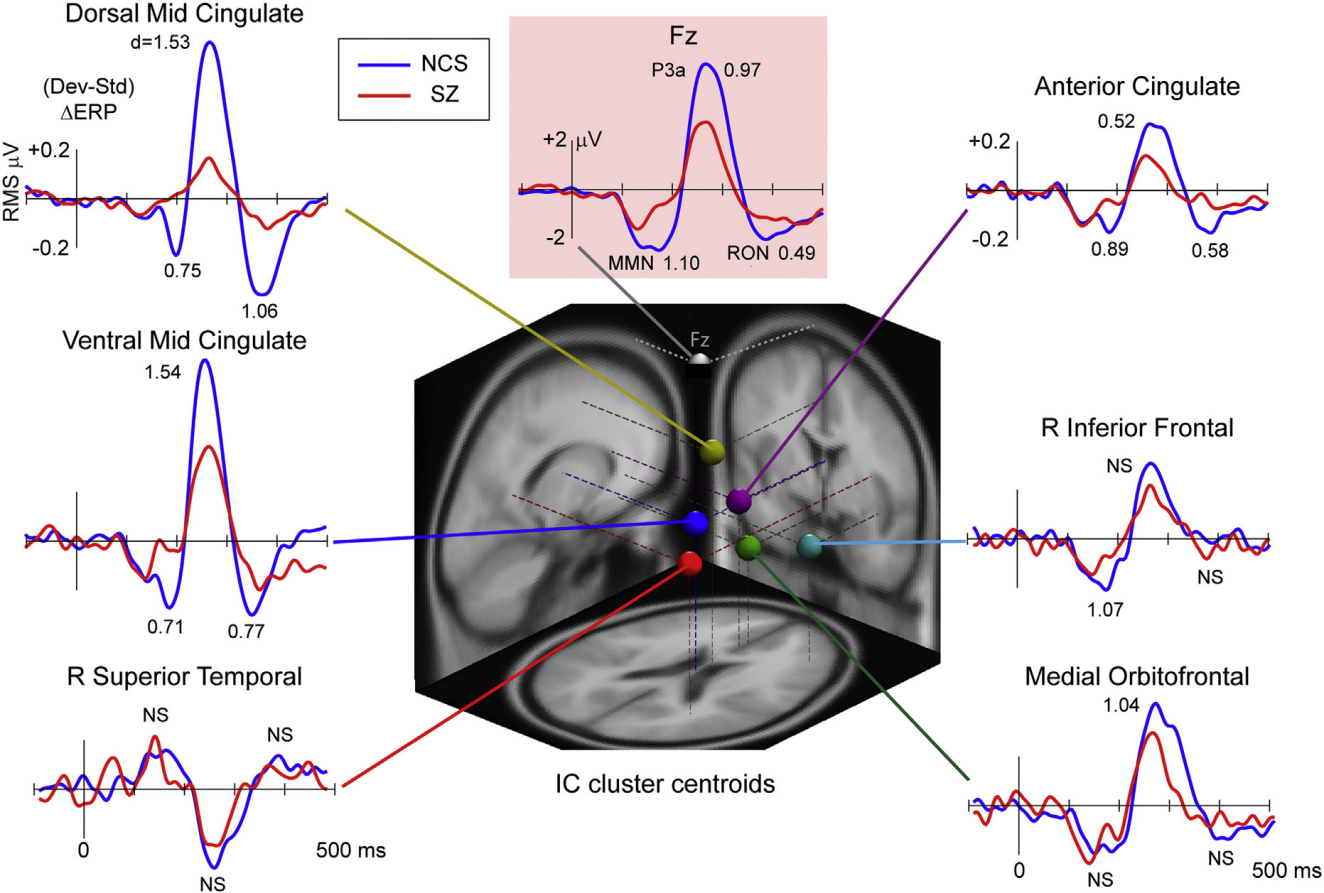
Grand-average deviance response waveforms for the NCS and SZ subject groups for frontocentral scalp channel Fz (highlighted in pink) and for the six contributing IC source clusters. The vertical scale for the source clusters is scalp-projected RMS µV across all scalp channels. The corresponding between-group Cohen's d effect sizes are noted (NS = non-significant) (center). The three-dimensional equivalent dipole localization plot shows the centroid locations of each IC source cluster in the MNI template brain.

Fig. 4 separates contributions of the six contributing IC clusters for the NCS and SZ subject groups. Group amplitude effect sizes (Cohen's d) are noted near each peak. For comparison, the group deviance responses at scalp channel Fz and effect sizes are also shown. Notably, two IC cluster effect sizes for P3a amplitude obtained for the independent component sources (ventral and dorsal mid-cingulate, d > 1.53) far exceed those obtained from the scalp sensor (Fz) data, although the Cohen's d value for the group effect for MMN peak amplitude at Fz (d = 1.10) was near the IC cluster effect size (R Inferior Frontal, d = 1.07).

### 3.4 Auditory deviance response peak latencies

**Fig. S1:**
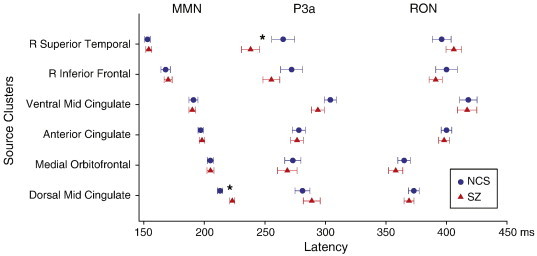
1 Mean Peak Latencies. Mean group source-resolved ERP-difference peak latencies (MMN, P3a, and RON). The order of the source clusters (left) is sorted by peak latencies of the maximal MMN contributions.

For MMN peak latency, a main effect of source cluster (F_5_,_374_=162.81, *p* < 0.0001) was present. Follow-up pair-wise contrasts within the primary model framework confirmed that the latency of the MMN peak in each cluster was significantly later than peak MMN latency in the preceding source cluster in the following order (all *F* > 6.90, all *p* < 0.01): R Superior Temporal, R Inferior Frontal, Ventral Mid-Cingulate, Anterior Cingulate, Medial Orbitofrontal, and Dorsal Mid-Cingulate cortex (Supplemental Figure 1). Analysis of source-resolved P3a and RON peak latencies revealed main effects of source cluster (*F* > 11.00, *p* < 0.0001) but no significant Group or Group-by-Cluster interactions.

Inline Supplementary Figure S1

### 3.5 Auditory deviance response peak amplitudes

In contrast to latency analyses, significant Group-by-Cluster interactions (all *F* > 3.00, all *p* < 0.01) were present for MMN, P3a, and RON peak amplitudes, each exhibiting significantly lower amplitudes in the SZ patients. Follow-up pair-wise contrasts within the primary model framework revealed significantly smaller MMN amplitude in SZ in the R Inferior Frontal (*F*1,373 = 9.38, *p* < 0.01), Ventral Mid-Cingulate (*F*1,347 = 5.60, *p* < 0.05), Anterior Cingulate (*F*1,332 = 11.94, *p* < 0.001), and Dorsal Mid-Cingulate (*F*1,329 = 9.64, *p* < 0.01) clusters. Smaller P3a amplitudes in SZ were present at Ventral Mid-Cingulate (*F*1,374 = 32.79, *p* < 0.0001), Anterior Cingulate (*F*1,374 = 6.00, *p* < 0.05), Medial Orbitofrontal (*F*1,374 = 11.17, *p* < 0.001), and Dorsal Mid-Cingulate (*F*1,374 = 55.94, *p* < 0.0001), while smaller RON amplitude deficits in SZ occurred in Ventral Mid-Cingulate (*F*1,322 = 10.72, *p* < 0.001), Anterior Cingulate (*F*1,307 = 9.56, *p* < 0.01), and Dorsal Mid-Cingulate (*F*1, 304 = 31.35, *p* < 0.0001) clusters.

### 3.6 Source cluster peak amplitude differences

Fig. 4 shows mean source cluster deviance response waveforms for the six identified source clusters. The units here are root mean-square (RMS) microvolts per source channel projection across the entire scalp montage. The [−0.2, +0.5] µV RMS scale of the individual component clusters here compares to the [0–2.0] µV RMS scale of the scalp channel response across all channels, as shown in Fig. 2 (bottom row).

### 3.7 Neurophysiological associations with clinical, cognitive and psychosocial measures

Table 3, Table 4 summarize significant (*r*^2^ > 10%) associations of MMN, P3a, and RON latencies and amplitudes with clinical, cognitive, and functional variables computed from the channel Fz deviance response as well as from the contributions of the most strongly contributing IC source clusters. Table 5 gives the (two-sided) *p*-values that correspond to the number of expected by chance alone versus number of observed correlations, stratified by *r*^2^ effect-size thresholds.

### 3.8 Peak latencies

Consistent with the majority of previous MMN, P3a, and RON studies, no significant functional measure correlations with peak latencies at electrode Fz were found. In contrast, twenty-two significant pairwise correlations (*r*^2^ ≥ 10%) were found between individual patient symptom and function scores and the peak latencies in the clustered IC responses, including sixteen correlations accounting for ≥20%, nine for ≥30%, three for ≥40%, and one for ≥50% of the across-subjects variance in the functional measures. Of these, eleven correlations (shown in bold) exceeded even the conservative Bonferroni significance thresholds.

### 3.9 Peak amplitudes

For peak amplitudes at scalp channel Fz only two weak (≥ 10%) correlations were observed, each explaining <13% of measure variance. As shown in Table 5, however, in this case 2.45 correlations ≥10% would be expected by chance alone. Peak amplitudes derived from source-resolved difference-response waveforms featured thirty significant correlations (uncorrected), including twenty that accounted for ≥20%, eleven for ≥30%, five for ≥40%, and one for ≥50% of the variance of the functional variable. Of these, fourteen correlations exceeded the conservative Bonferroni significance thresholds (shown in bold).

## 4 Discussion

This study used a source decomposition approach applied to the whole-EEG signals to identify the cortical IC source signals and areas underlying the group differences in auditory deviance responses in schizophrenia patients and normal control subjects in a paradigm in which participants were instructed to concentrate on an entertaining video rather than on the concurrently presented tone stimulus stream. We identified a network of not two but six cortical independent source domains, distributed across medial and frontotemporal cortex, that contributed most strongly to the auditory deviance response.

Each of the six IC clusters produced a triphasic auditory deviance response complex, here measuring the mean response difference evoked by occasional (10%) slightly longer (100-ms) Deviant tones interspersed in a sequence of (shorter 50-ms) Standard tones. Thus, no source cluster contributed to only one of the (MMN, P3a, or RON) peak sequence of the auditory deviance response. Individual peak measures of the source domain contributions to the scalp-recorded deviance response exhibited robust and biologically plausible relationships with many SZ-subject measures in the clinical, cognitive, and psychosocial domains. Notably, this was unlike equivalent measures computed on the most indicative (Fz) scalp channel signal itself. The relative strength of the source-resolved measure correlations is compatible with the biophysical fact that each scalp channel recording sums contributions from many cortical areas, both relevant and irrelevant.

The six source clusters contributing in both NCS and SZ patients to the triphasic deviance response complex at frontocentral scalp channels were centered in or near: 1) R Superior Temporal, 2) R Inferior Frontal, 3) Ventral Mid-Cingulate, 4) Anterior Cingulate, 5) Medial Orbitofrontal, and 6) Dorsal Mid-Cingulate cortex. MMN peak latencies in the six clusters varied between 153 and 223 ms. Analyses of source-projected waveforms revealed near-equal contributions to the MMN, P3a, and RON peaks from right superior temporal sources in SZ patients and controls, with varying reductions in peak amplitudes in SZ across the remaining five source clusters (Fig. 4). Specifically, diminished peak amplitudes were present in Ventral Mid-Cingulate, Anterior Cingulate, and Dorsal Mid-Cingulate source clusters for MMN (d = 0.70 to 1.07); Ventral Mid-Cingulate, Anterior Cingulate, Medial Orbitofrontal, and Dorsal Mid-Cingulate source clusters for P3a (d = 0.52 to 1.54); and Ventral Mid-Cingulate, Anterior Cingulate, and Dorsal Mid-Cingulate source clusters for RON (d = 0.58 to 1.06). Overall, group amplitude differences were most marked and significant for the Ventral and Dorsal Mid-Cingulate clusters (Fig. 4, upper left).

### 4.1 Comparison to previous reports

Collectively, results of these AMICA-based decompositions of the whole EEG extend and refine previous studies reporting that MMN generators must be broadly distributed across primary and secondary auditory cortices (Alho, 1995; Frodl-Bauch et al., 1997; Jääskeläinen et al., 2004; Jemel et al., 2002; Kropotov et al., 1995; Molholm et al., 2005; Takahashi et al., 2012; Tiitinen et al., 1993) and are followed by P3a and RON contributions across frontal sources (Jemel et al., 2002; Marco-Pallares et al., 2005; Oknina et al., 2005; Rinne et al., 2000; Schönwiesner et al., 2007; Takahashi et al., 2012; Waberski et al., 2001; Wild-Wall et al., 2005). These results also confirm findings of a larger MMN contribution from right versus left hemisphere (Paavilainen et al., 1991). In this study, deficits in SZ patients were more pronounced in frontomedial (Takahashi et al., 2012) rather than the commonly assumed temporal sources (Umbricht and Krljes, 2005), likely because of the duration mismatch design used here in which the deviance of the Deviant tones was marked by the *absence* of expected tone *offset* at 50 ms after stimulus onset rather than by frequency or intensity differences occurring at tone onset. For this reason, MMN peak latencies in this study were, as should be expected, later than those obtained in studies using other types of auditory deviance.

The absence of source clusters in left and right auditory cortices from the six source clusters identified as contributing to the auditory deviance response deserves comment. We here focused only on the source clusters making the largest differential contributions to the recorded deviant and standard stimulus responses. Left and right auditory cortical clusters did appear among our 21 source clusters, and both made clear contributions to the early auditory ERPs. But both clusters also produced very similar responses to standard and deviant stimuli. Again, we believe this likely arose from the auditory duration deviance protocol we employed in which the deviance feature (delayed tone offset) was not available in the first 50 ms the stimulus was sounding as it would be in other auditory deviance paradigms.

### 4.2 Individual subject differences

Importantly, the source-resolved auditory deviance response peak measures for these data exhibited significant correlations with clinical, cognitive, and psychosocial characteristics of the individual SZ patients (Table 3, Table 4), accounting for 10–50% of the variance in several of these measures. The number, magnitude, and pattern of these associations suggest that they are unlikely to be specious. We took great care to control for Type I error (Table 5), but twenty-five significant source-level ERP correlations exceeded even the stringent Bonferroni significance threshold, far more than the under three expected by chance alone.

While these exploratory results from a limited population sample restrict extensive interpretation, their physiological plausibility is supported by a variety of evidence. For example, frontal source activations reflect the recruitment of distributed attentional networks previously associated with executive functions (Szeszko et al., 2000) and cognitive control (Derrfuss et al., 2004; MacDonald et al., 2000) including the detection of salient stimuli (R Inferior Frontal; Hampshire et al., 2010; Hampshire et al., 2009); error detection and monitoring (Anterior Cingulate; Bush et al., 2000; Carter et al., 1998; MacDonald et al., 2000), response inhibition (R Inferior Frontal, Medial Orbitofrontal; Aron et al., 2003; Chikazoe et al., 2007; Goldstein et al., 2001), and updating working memory (Mid-Cingulate; Courtney et al., 1997; Nyberg et al., 2003). The dorsal mid-cingulate cluster location and its triphasic response strongly resemble those of a source cluster found to be a causal hub in a cortical network response to self-realized errors in an Ericksen flanker task underlying the Error-Related Negativity (ERN) in normal subjects (Mullen et al., 2010; Mueller et al., 2011; McLoughlin et al., 2014b). These relationships will be examined in more detail in future analyses of a much larger participant cohort.

The finding that right superior temporal cluster MMN amplitude accounted for 48% of the variance in tasks necessary for independent functioning extends previous reports of correlations between smaller MMN at one or more scalp electrodes and functional impairments (Kawakubo and Kasai, 2006; Light, and Braff 2005a, Light, and Braff 2005b; Light et al., 2007; Rasser et al., 2011; Wynn et al., 2010). In contrast to previous reports that relied on scalp sensors and clinician ratings of global functioning, the current study demonstrates, for the first time, relationships between EEG source measures and the UPSA (Patterson et al., 2001), a highly reliable (Light et al., 2012; Velligan et al., 2014) and well-validated performance-based measure of everyday functional capacity that is considered the standard in psychiatric research (Harvey, 2014; Harvey et al., 2010; Harvey et al., 2009; Mausbach et al., 2008; Mausbach et al., 2011). Likewise, the finding that Medial Orbitofrontal P3a amplitude accounted for 40% and 54% of the variance in positive and negative symptoms, respectively, is not inconsistent with reports using scalp electrode measures (Mathalon et al., 2000; Turetsky et al., 1998) and provides further validation of the links between sensory processing impairments and clinical characteristics of the SZ patients.

Notably, deviance response RON peak latency and P3a peak amplitude and latency for the Medial Orbitofrontal cluster loaded strongly onto Positive and Negative Symptoms, Executive and Psychosocial Functioning, and Functional Capacity scale differences between SZ subjects, making it of future interest for clarifying brain dynamic differences underlying the broad landscape of individual differences in SZ symptoms and functioning. By contrast, ERP peak measures for the IC Cluster with the largest group MMN amplitude effect size (Right Inferior Frontal) showed strong correlation with differences in cognitive abilities between SZ subjects (Auditory Attention, Working Memory, and Verbal IQ).

Separate exploratory analyses of the normal control subject group data also showed some correlations between deviance response peak measures and cognitive ability scores. We plan to examine these relationships in more detail in future analyses of a larger participant cohort.

Clearly, testing for correlations between individual peak measures and individual clinical variables for this subset of our much larger dataset is only a first step toward modeling the interactions between clinical status and the full ERP time courses as well as other measures of EEG data from the auditory deviance response paradigm. Planned future steps will include use of canonical correlation and non-linear machine learning methods as well as source-resolved causal network analysis (Mullen et al., 2010) to assess which cortical areas drive response activity in other areas.

### 4.3 Single-subject versus group ICA

The scalp maps and source locations of some of our identified source clusters resemble those reported earlier (Marco-Pallares et al., 2005) in a study of 30-channel EEG data collected in healthy subjects in a passive duration oddball paradigm. In that study, after subtracting the subject-mean standard-tone response from each deviant-tone epoch, deviant-tone epochs were concatenated across subjects and decomposed by Infomax ICA. This and other ‘group ICA’ analysis methods have the drawback of forcing a single decomposition of data from subjects with different head shapes, cortical source orientations, and resulting scalp projection pattern differences. The individual subject ICA decomposition and across-subject ICA clustering method we used here avoid this simplification, in principle allowing both more accurate spatial localization and time course estimation in individual subjects (Tsai et al., 2014), although at the expense of not forcing a solution on all subjects, thus allowing ‘missing data’ in each cluster from (as here) a substantial number of subjects.

In future work, we plan to address this problem by testing the information value of using joint group and single-subject ICA decompositions to obtain source cluster activity estimates for all subjects. However, the true nature of individual differences in EEG source distribution and dynamics is still unknown, so any assumption of subject uniformity should be applied with caution. Also, source clustering using somewhat a different method (Bigdely-Shamlo et al., 2013) should be of interest to apply to these data and the larger dataset they are drawn, and source clustering not in 3-D brain volume space but in cortical surface estimates have also been demonstrated and may be advantageous in cases when magnetic resonance (MR) head images are available for all participants (Tsai et al., 2014).

### 4.4 Use of ICA in clinical EEG studies

In recent years, approaching two decades after the first use of ICA for analysis of EEG (Makeig et al., 1996) and fMRI (McKeown and Sejnowski, 1998) data analysis, ICA methods have become widely used in neuroscience for identifying distinct dimensions of neurophysiological data of various types, both for artifact removal and for the identification of information-bearing brain source signals and source networks in clinical group EEG studies. For some researchers long accustomed to standard EEG scalp-channel measures, the ICA source decomposition approach may represent a challenging paradigm shift, particularly as long-term examination of scalp-channel measures alone might prompt some researchers to in practice imagine (if not believe) that the recorded signals represent brain potentials that flow directly upwards from the cortical surface to the supervening scalp electrodes. Biophysical theory and measurements, however, support quite a different model wherein local currents that are spatially coherent or near coherent across a (more or less cm^2^-scale) cortical domain or patch are volume conducted to nearly all the scalp electrodes. Small coherent signal domains have a much smaller scalp (or far field) projection and, because of their larger number and close spacing, tend to phase-cancel each other's scalp projections.

Each scalp-channel signal is, therefore, a mixture of contributions of varying strengths from a finite number of distinct (cm^2^-scale or larger) source processes located across the cortex as well as a variety of non-brain (‘artifact’) source processes (Makeig et al. 1996, Makeig et al. 1997). ICA decomposition minimizes the strong ‘mixing’ effects of common volume conduction from these cortical and other non-brain EEG source processes to each scalp electrode and subsequent summation of source signals in the scalp channel signals. When applied to a sufficient amount of multi-channel EEG data, ICA decomposes the data into distinct brain and non-brain artifact sources (plus, typically, a low-amplitude, not further definable ‘noise’ subspace). The benefit of ICA applied to EEG data is that it identifies maximally distinct sources of *information* in the EEG data — and these are found to have physically separable physical origins and typically exhibit functional independence (Makeig et al., 2004; Makeig & Onton, 2011).

Further, ICA decomposition returns the projection pattern of each source to the scalp montage (typically visualized as an interpolated IC scalp map), thereby also greatly reducing the complexity and under-determination of the source localization problem, since in this case a single equivalent dipole model can be used to define the approximate cortical source location (Acar and Makeig, 2013) and, when a participant magnetic resonance head image is available, the more exact location of the generating cortical patch (Acar et al., 2011; Tsai et al., 2014).

As with most new methods, some cautions are in order. First, as a ‘blind’ separation technique, ICA cannot be guaranteed to yield physiologically meaningful results, particularly when the data to be decomposed are in some way insufficient. The quality of ICA decomposition can vary considerably with the amount and suitability of the input data and even with the particular ICA algorithm used, though applied to enough data of high enough quality, ICA results from the same or different algorithms are typically similar (Delorme et al., 2012). However, not every independent component is equally statistically robust or physiologically plausible; restricting the data analysis to IC processes compatible with a plausibly localized cortical source is typically most fruitful (Delorme and Makeig, 2004).

Second, this approach is computationally intensive and until recently impractical to implement on smaller computers. Our analyses required over 4 h per subject of continuous run time on a 64-processor cluster to perform the initial AMICA decomposition. These analyses, therefore, might have required over 2 years of continuous run time on a single-core processor. However, ongoing refinements to open-source software can offload computational demands to high-end video cards (graphic processing units, now commonly used for computer gaming), and deliver a many-fold reduction in processing time, allowing routine application of these methods using inexpensive personal computers (Raimondo et al., 2012). Future analyses might explore and compare additional source modeling approaches including Dynamic Casual Modeling for the analysis of temporal and spatial EEG data (Penny et al., 2011), for which the present ICA-derived results might provide a viable test model.

### 4.5 Effects of medications

Lastly, as is often the case for studies of schizophrenia, medications were not experimentally controlled in this study. Although no significant cross-sectional antipsychotic medication effects are detected in scalp-recorded ERPs in this paradigm, as detailed in Rissling et al. (2012) using identical stimulation procedures, the potential effects of psychoactive medications on cortical source activations or on the functional balance of sources have not been investigated. The substantial heterogeneity of antipsychotic and adjunctive treatments, variable degree of adherence to prescribed medication regimens, and multiple pathways to receiving prescriptions of particular drugs (e.g., given insurance limits on access to specific treatments) greatly complicate attempts to disentangle potential medication effects. Prospective randomized controlled studies are needed to clarify the impact, if any, of antipsychotic medications on source-resolved neurophysiological measures and/or their clinical symptom and cognitive scale correlations.

More generally, these results demonstrate the utility of applying advanced source-level data decomposition, if desired via open source software (here, EEGLAB and its extensions, available at http://sccn.ucsd.edu/eeglab/), to whole EEG signals collected in clinical and other studies. Expanded use of EEG source imaging tools could make possible far-reaching applications in neuroscience and neuropsychiatry. For example, source-resolved neurophysiological measures may provide endophenotypes in genomic studies and sensitive biomarkers for subject classification and might be used to predict and monitor responses to clinical interventions (Braff and Light, 2004; Lenartowicz et al., 2014; Light and Näätänen, 2013; Light and Swerdlow, in press; McLoughlin et al., 2014a; Perez et al., 2014).

## Conflict of interest

Dr. Light has served as a consultant for Envivo, Astellas, and Neuroverse, Inc. for matters unrelated to this study. The remaining authors report no conflicts of interest.

## Figures and Tables

**Table 1:** Demographic and clinical characteristics of the non-psychiatric comparison and schizophrenia patient groups (means ± SD given where applicable).

Demographic and clinical characteristics	Schizophrenia patients (*n* = 42)		Non-psychiatric comparison subjects (*n* = 47)	
Mean	SD	Mean	SD
*Gender (% male)	63.41	−	44.89	−
Age (years)	45.36	9.58	42.99	11.93
Years of education completed	11.90	1.95	14.48	2.20
Age of illness onset	19.72	4.53	−	−
Duration of illness	23.63	9.02	−	−
Number of hospitalizations	7.15	6.81	−	−
SANS total score	14.41	4.82	−	−
SAPS total score	8.68	4.50	−	−
GAF total	40.80	4.77	−	−
SOF total	47.12	6.05	−	−
UPSA total	81.25	11.04	−	−

Abbreviations: SANS, Scale for the Assessment of Negative Symptoms; SAPS, Scale for the Assessment of Positive Symptoms, UPSA, (University of California San Diego) Performance Based Skills Assessment.

* The proportion of men to women in each group was significantly different χ^2^ = 8.64, *p* < 0.01.

**Table 2:** Antipsychotic medication characteristics of the schizophrenia patient group.

Antipsychotic medication	Mean dose (mg)	Range (mg)
Olanzapine (*N* = 11)	15	4–30
Risperidone (*N* = 10)	3	1–8
Quetiapine (*N* = 9)	344	200–600
Clozapine (*N* = 8)	328	100–450
Ziprasidone (*N* = 5)	89	20–180
Aripiprazole (*N* = 3)	13.33	10–15
Chlorpromazine (*N* = 2)	200	−
Fluphenazine (*N* = 2)	32	15–50
Paliperidone (*N* = 1)	9	−

**Table 3:** Amplitude correlations. Summary of associations among scalp electrode Fz and source-resolved ERP amplitudes with clinical, neurocognitive and functional variables in schizophrenia patients. Correlations shown in bold exceed two-tailed Bonferroni significance level adjustments (Fz: α = 0.05/30 = 0.002, *r*^2^ > 0.22; source-resolved ERPs: α = 0.05/180 = 0.0003; *r*^2^ > 0.28). Number of significant correlations: Fz: uncorrected = 2, Bonferroni = 0; source resolved ERPs: uncorrected = 30, Bonferroni = 14.

	ERP	*r*^2^
Scalp electrode (Fz)		
Verbal IQ (WRAT)	P3a	0.11
Functional capacity (UPSA)	RON	0.12
R Superior temporal		
Working memory (LNS reorder)	RON	0.15
Verbal IQ (WRAT)	RON	0.15
**Immediate verbal memory (CVLT)**	**RON**	**0.28**
Delayed verbal memory (CVLT)	RON	0.26
**Functional capacity (UPSA)**	**MMN**	**0.48**
Functional capacity (UPSA)	RON	0.26
R inferior frontal		
**Negative symptoms (SANS)**	**RON**	**0.36**
Psychosocial functioning (SOF)	RON	0.24
**Auditory attention (LNS forward)**	**MMN**	**0.38**
**Working memory (LNS reorder)**	**MMN**	**0.30**
**Verbal IQ (WRAT)**	**MMN**	**0.46**
Ventral mid-cingulate		
**Positive symptoms (SAPS)**	**RON**	**0.29**
**Negative symptoms (SANS)**	**P3a**	**0.36**
**Immediate verbal memory (CVLT)**	**RON**	**0.41**
Delayed verbal memory (CVLT)	RON	0.24
**Verbal IQ (WRAT)**	**RON**	**0.29**
Executive functioning (WCST)	RON	0.24
Anterior cingulate		
Functional status (GAF)	MMN	0.18
Functional status (GAF)	RON	0.17
Immediate verbal memory (CVLT)	RON	0.25
Delayed verbal memory (CVLT)	RON	0.17
Medial Oribitofrontal		
**Positive symptoms (SAPS)**	**P3a**	**0.40**
**Negative symptoms (SANS)**	**P3a**	**0.54**
**Psychosocial functioning (SOF)**	**P3a**	**0.37**
**Functional capacity (UPSA)**	**P3a**	**0.32**
Dorsal mid-cingulate		
Verbal IQ (WRAT)	P3a	0.15
Executive functioning (WCST)	MMN	0.18

**Table 4:** Latency correlations: Summary of associations among scalp electrode Fz and source-resolved ERP latencies with clinical, neurocognitive and functional variables in schizophrenia patients. Correlations shown in bold exceed two-tailed Bonferroni significance level adjustments (Fz: α = 0.05/30 = 0.002, *r*^2^ > 0.22; source-resolved ERPs: α = 0.05/180 = 0.0003; *r*^2^ > 0.28). Number of significant correlations: Fz: uncorrected = 0, Bonferroni = 0; source-resolved ERPs: uncorrected = 22, Bonferroni = 11.

	ERP	*r*^2^
Scalp electrode (Fz)		
---n/a---	−	−
R superior temporal		
Functional capacity (UPSA)	MMN	0.25
Delayed verbal memory (CVLT)	MMN	0.17
R inferior frontal		
**Negative symptoms (SANS)**	**RON**	**0.51**
Psychosocial functioning (SOF)	RON	0.25
**Executive functioning (**WCST**)**	**MMN**	**0.30**
**Executive functioning (**WCST**)**	**P3a**	**0.28**
Ventral mid-cingulate		
**Negative symptoms (SANS)**	**P3a**	**0.33**
**Negative symptoms (SANS)**	**RON**	**0.33**
**Psychosocial functioning (SOF)**	**P3a**	**0.31**
Verbal IQ (WRAT)	MMN	0.25
**Executive functioning (**WCST**)**	**P3a**	**0.30**
Anterior cingulate		
Functional capacity (UPSA)	RON	0.17
Verbal IQ (WRAT)	MMN	0.24
Auditory attention (LNS-Forward)	MMN	0.17
Medial orbitofrontal		
**Negative symptoms (SANS)**	**RON**	**0.41**
**Positive symptoms (SAPS)**	**RON**	**0.40**
**Auditory attention (LNS-forward)**	**MMN**	**0.29**
**Executive functioning (**WCST**)**	**P3a**	**0.32**
Dorsal mid-cingulate		
Negative symptoms (SANS)	MMN	0.20
Negative symptoms (SANS)	P3a	0.17
Global functioning (GAF)	RON	0.24
Functional capacity (UPSA)	P3a	0.13

**Table 5:** Summary of expected (based on chance alone) and observed clinical variable/ERP-peak measure correlations for schizophrenia patients, stratified by magnitude of *r*^2^ effect sizes. Bonferroni-adjusted critical *p*-values (2-sided) are shown for scalp-channel average ERPs at electrode Fz and for the 6 source clusters, pooled across measures of ERP peaks MMN, P3a and RON.

*r*^2^	Adjusted critical *p*-value	Expected # significant correlations		Observed # significant correlations (amplitude)		Observed # significant correlations (latency)	
Traditional ERP	Source resolved ERP	Traditional ERP	Source resolved ERP	Traditional ERP	Source resolved ERP
>10%	0.05	2.45	14.69	2	30	0	22
≥20%	0.008	0.29	1.73	0	20	0	16
≥30%	0.0008	0.03	0.17	0	11	0	9
≥40%	0.00006	0.002	0.01	0	5	0	3
≥50%	0.000003	0.000003	0.0006	0	1	0	1

**Table 6:** Breakdown of the number of independent components (#ICs) and the number of subjects (#Ss) per group contributing to each contributing source cluster.

	NCS		SZ	
Source cluster	#ICs	#Ss (of 47)	#ICs	#Ss (of 42)
R superior temporal	42	32	37	30
R inferior frontal	14	14	18	18
Ventral mid-cingulate	32	25	25	17
Anterior cingulate	45	30	42	30
Medial orbitofrontal	42	34	53	34
Dorsal mid-cingulate	27	20	16	15
*Means*	33.7 (1.31/S)	25.8 (55%)	31.8 (1.33/S)	24 (57%)

## Footnotes

1 See sccn.ucsd.edu/wiki/Chapter_05:_Component_Clustering_Tools#Preparing_to_cluster_.28Pre-clustering.29_with_PCA.28 original.29.
