# Supplementary material for: Cortical substrates and functional correlates of auditory deviance processing deficits in schizophrenia
Source: Neuroimage Clin. 2014 Oct 1;6:424–37. doi: 10.1016/j.nicl.2014.09.006 (PMC4218942; doi:10.1016/j.nicl.2014.09.006)
Supplement: Inline Supplemental Table 1 [file mmc1.docx]

**Table 1 (Supplemental): Latency Correlations.** Summary of associations among scalp electrode Fz and source-resolved ERP latencies with neurocognitive variables in nonpsychiatric comparison subjects. Correlations shown in bold exceed two-tailed Bonferroni significance level adjustments (Fz; α=0.05/36 = 0.002; r^2^ values > 0.26), (source-resolved ERPs; α =0.05/216= 0.0002; r^2^ values > 0.32). Number of significant correlations: (Fz) uncorrected=2, Bonferroni=2, source resolved ERPs uncorrected=6, Bonferroni=1.

|  | **ERP** | **r^2^** |
| --- | --- | --- |
| Scalp Electrode (Fz) |  |  |
| Working Memory (LNS Reorder) | **MMN** | **0.24** |
| Working Memory (LNS Reorder) | **P3a** | **0.27** |
| R Superior Temporal |  |  |
| Working Memory (LNS Reorder) | MMN | 0.27 |
| Executive Functioning (WCST) | RON | 0.29 |
| R Inferior Frontal |  |  |
| **Auditory Attention (LNS Forward)** | **RON** | **0.52** |
| Ventral Mid Cingulate |  |  |
| ---n/a--- |  |  |
| Anterior Cingulate |  |  |
| Immediate Verbal Memory (CVLT) | P3a | 0.15 |
| Medial Oribitofrontal |  |  |
| ---n/a--- |  |  |
| Dorsal Mid Cingulate |  |  |
| Executive Functioning (WCST) | P3a | 0.24 |
| Executive Functioning (WCST) | RON | 0.24 |
