# Supplementary material for: Cortical substrates and functional correlates of auditory deviance processing deficits in schizophrenia
Source: Neuroimage Clin. 2014 Oct 1;6:424–37. doi: 10.1016/j.nicl.2014.09.006 (PMC4218942; doi:10.1016/j.nicl.2014.09.006)
Supplement: Inline Supplemental Table 3 [file mmc3.docx]

| **r^2^** | ***Adjusted Critical***  ***p-value*** | ***Expected # Significant Correlations*** | | ***Observed # Significant Correlations***  ***(Amplitude)*** | | ***Observed # Significant Correlations***  ***(Latency)*** | |
| --- | --- | --- | --- | --- | --- | --- | --- |
|  |  | **Traditional ERP** | **Source Resolved ERP** | **Traditional ERP** | **Source Resolved ERP** | **Traditional ERP** | **Source Resolved ERP** |
| >10% | .037 | 0.89 | 5.34 | 2.0 | 6.0 | 2.0 | 6.0 |
| ≥20% | .0023 | 0.06 | 0.33 | 2.0 | 3.0 | 2.0 | 5.0 |
| ≥30% | .00012 | 0.003 | 0.02 | 0.0 | 1.0 | 0.0 | 1.0 |
| ≥40% | .000004 | 0.0001 | 0.0006 | 0.0 | 0.0 | 0.0 | 1.0 |
| ≥50% | .00000008 | 0.000002 | 0.00001 | 0.0 | 0.0 | 0.0 | 1.0 |

**Table 3 (Supplemental).** Summary of expected (based on chance alone) and observed correlations stratified by magnitude of r^2^effect sizes in nonpsychiatric comparison subjects. Bonferroni adjusted critical *p*-values (2-sided) for traditional ERP averaging at electrode Fz and the combined 6 sources pooled across MMN, P3a and RON ERPs are shown.
